# Water quality, human health risk, and pesticides accumulation in African catfish and Nile tilapia from the Kitchener Drain-Egypt

**DOI:** 10.1038/s41598-023-45264-3

**Published:** 2023-10-28

**Authors:** Ahmed A. Metwally, Malik M. Khalafallah, Mahmoud A. O. Dawood

**Affiliations:** 1 Department of Aquaculture, Faculty of Aquatic and Fisheries Sciences, Kafrelsheikh University, Kafr El-Shaikh, 33516 Egypt; 2 Animal Production Department, Faculty of Agriculture, Kafrelsheikh University, Kafr El-Shaikh, 33516 Egypt; 3 The Center for Applied Research on the Environment and Sustainability, The American University in Cairo, Cairo, 11835 Egypt

**Keywords:** Biochemistry, Zoology, Environmental sciences, Natural hazards

## Abstract

Pesticides are toxic and could negatively impact humans and the ecosystem. The Kitchener Drain is among the longest drains in Egypt and carries a wide range of wastewater from the agriculture sector, which contains pesticides and may pollute the ecosystem. Thus, water quality, human health risk, and pesticide accumulation in African catfish and Nile tilapia from the Kitchener Drain-Egypt. The water and fish samples were collected from Kitchener Drain in Kafr Elsheikh Governorate, Egypt, during the four seasons. The results indicated that heptachlor and diazinon were undetected during the four seasons. However, endosulfan, chlorpyrifos, and dicofol were detected in winter and autumn. Only p,p′-DDT was detected during spring. Endosulfan, heptachlor, and aldrin were detected in Nile tilapia during winter. Only heptachlor and aldrin were detected during spring. Endosulfan, heptachlor, dicofol, p,p′-DDT, chlorpyrifos, and diazinon were detected in the autumn season. In summer, dicofol and p,p′-DDT were detected, while endosulfan, heptachlor p,p′-DDT, aldrin, chlorpyrifos, and diazinon were not detected. In African catfish, endosulfan, heptachlor, dicofol, and p,p′-DDT were detected during winter, while chlorpyrifos, aldrin, and chlorpyrifos, aldrin, and diazinon were not detected. In the spring season, endosulfan, heptachlor, and aldrin were detected. Endosulfan, heptachlor, dicofol, p,p′-DDT, aldrin, chlorpyrifos, and diazinon were detected in the autumn season. Similarly, in the summer season, endosulfan, heptachlor, dicofol, p,p′-DDT, aldrin, chlorpyrifos, and diazinon were detected. The sequence of estimated daily intake (EDI) in Nile tilapia during the four seasons is heptachlor > endosulfan > dicofol > p,p′-DDT > aldrin > diazinon > chlorpyrifos. The sequence of EDI in African catfish during the four seasons is endosulfan > p,p′-DDT > heptachlor > aldrin > dicofol > diazinon > chlorpyrifos. In conclusion, the results confirmed the absence of a hazard index for consuming Nile tilapia and African catfish collected from the Kitchener drain.

## Introduction

A considerable number of pesticides and insecticides are used in the agriculture sector^1^. Directly or indirectly, these toxicants' derivatives reach the water bodies and induce negative impacts on the ecosystem^2^. Many studies tackled the impacts of accumulated pesticides and their derivatives on water drains, sediments, and living organisms, especially fish^3^. The hazards of water pollution with pesticides affect human health since accumulated derivates could reach the human body through the water or fish as food^4^. Fish are utilized as bioindicators in case of high pollution in the water bodies due to their low digestion capacity for the accumulated pesticides^5–7^. Pesticides and toxicants can accumulate in the fish's body through the gills, skin, and intestines^8^.

Pesticides are known for their toxic effects on humans and the ecosystem due to their long persistence, high bioaccumulation capacity, and long-range transport^9^. Organochlorine and organophosphorus pesticides are heavily used in agriculture, and their derivates could pollute the water bodies and cause severe impacts^10^. Besides, organochlorine and organophosphorus compounds are known for their toxic effects on humans and the ecosystem due to their long persistence, high bioaccumulation capacity, and long-range transport^9^. Further, the sediments and some fish species, such as Nile tilapia and African catfish, are rich in organochlorine and organophosphorus derivates and may have human health risk^11, 12^. One of the famous drains in the northern area of Egypt is the Kitchener drain which collects water from the agricultural and industrial sectors^13^. More specifically, the existing section of the Kitchener drain in Kafrelsheikh governorate, which is famous for agriculture and fish farming activities. Nile tilapia is a known species worldwide and can grow under farming conditions or in the wild^14^. Another well-known fish species, famous for its delectable taste, is the African catfish which prefers to live in the bottoms of water bodies. This results in a high possibility of organochlorine and organophosphorus accumulation through the sediments^15^. Both fish species thrive in the Kitchener drain and can be captured to be introduced into the human food chain. Therefore, the human food chain is expected to get polluted with pesticide derivates through the water and fish. Hence, this study was conducted to test the presence of organochlorine and organophosphorus in the Kitchener drain using Nile tilapia and African catfish as bioindicators. Further, the pesticides were tested in the muscles of Nile tilapia and African catfish, as well as the human risk assessment.

Pesticide pollution is increasingly spreading, especially in developing countries, due to the lack of awareness and regulations^16^. Egypt is famous for agricultural activity, which depends on using a wide range of pesticides. Nevertheless, enormous amounts of pesticides threaten the water bodies, and consequently, their waste pollutes the drainage water^17^. Kitchener drain is a central drain that crosses through several vital crowdy cities in the Delta area. Its water is full of pesticides and industrial waste that can reach the human food chain and cause severe health risks^11, 18^. The aquatic system is also expected to suffer from the pollution of pesticides that may accumulate in the fish's edible tissues^19^. The bioaccumulation of pesticides was detected earlier in some famous consumed fish species in Egypt, such as Nile tilapia and African catfish^11, 12^. Hence, this study tested the presence of organochlorine and organophosphorus in the Kitchener drain using Nile tilapia and African catfish as bioindicators. Further, the pesticides were tested in the muscles of Nile tilapia and African catfish and the human risk assessment.

## Materials and methods

### Site location and sampling

The Kitchener Drain is among the longest in Egypt, with a total of 69 km, and passes through three governorates in the Delta area (Dakahlia, Gharbia, and Kafr El-Sheikh). Forty-six kilometers of the Kitchener Drain is located inside Kafr El-Sheikh governorate before draining the water to the Mediterranean Sea. El-Burullus lagoon and surrounding areas located in Baltim city in Kafr El-Sheikh are famous for aquaculture-related activities where the water in Kitchener Drain is used for fish farming.

**Figure 1:**
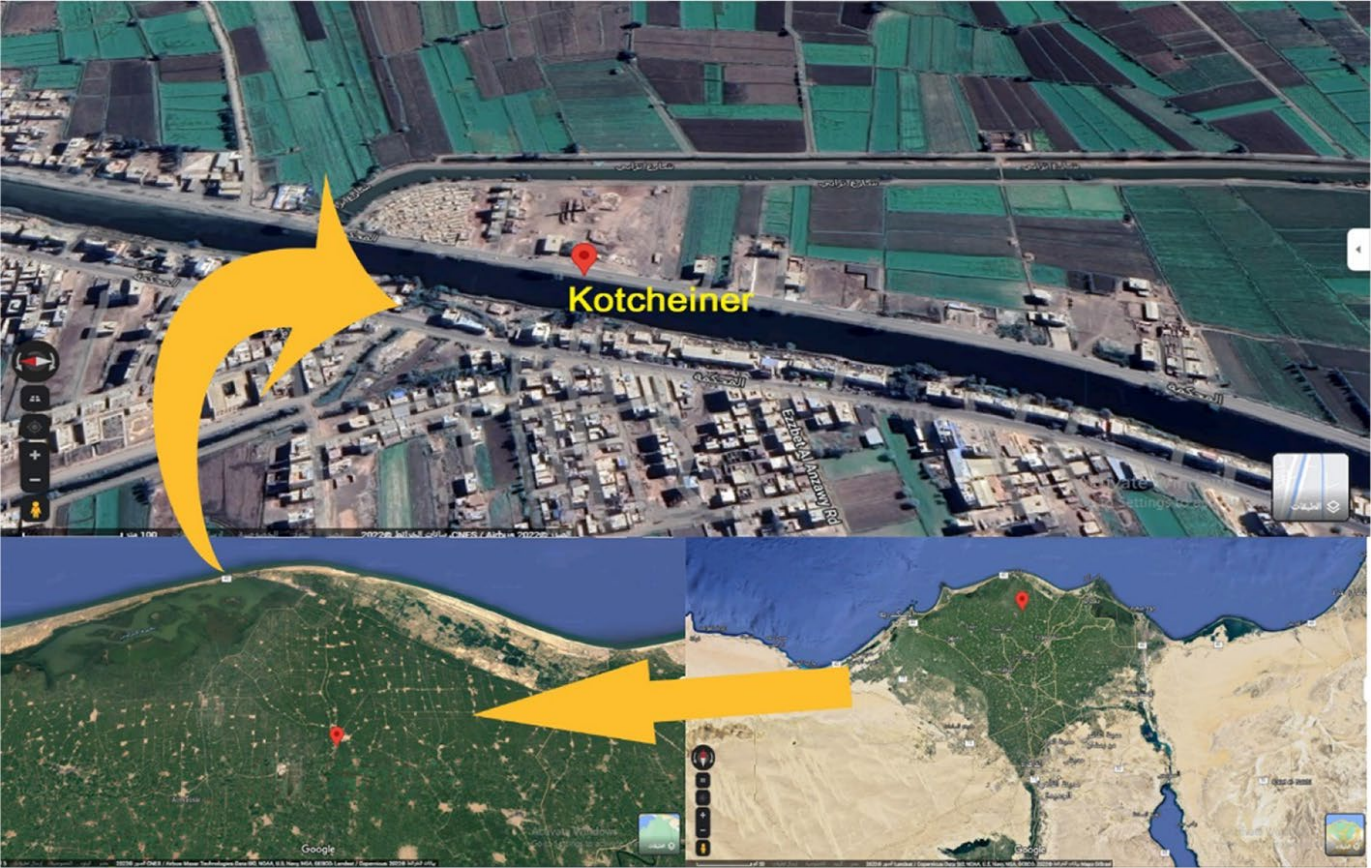
The sampling site for fish species and water samples were assessed in this study (Satellite image created by using Google Earth Pro version 7.3.0.3832 https://www.neowin.net/news/google-earth-pro-7303832/).

The samples were collected from Kitchener Drain in Kafr Elsheikh Governorate, between longitudes 31° 08′ 25.6′′ E and latitudes 31° 18′ 02.4′′ N (Fig. 1). Water and fish samples were collected from the Kitchener Drain during four seasons from April 2021 to March 2022. The samples were collected in May (spring), August (summer), November (autumn), and February (winter). Two fish species were collected from Kitchener Drain (Nile tilapia (*Oreochromis niloticus*) and African catfish (*Clarias gariepinus*)) were collected. Fish was collected and kept in an ice box at 4 °C. Subsequently, water samples were collected 20 cm below the water surface and kept in sterile water containers while treated with 1 mL of HCL to eliminate microbial activity.

### Water samples and analysis

The collected water samples were filtered through 0.45 μm fiberglass filters (Whatman) to remove suspended materials. Water samples were collected and kept in 500 mL sterile plastic containers. After filtration, samples were stored in dark glass vials for GC determination. Water samples were divided into two parts, the first part for determining the water’s physical parameters and the second part for analysis of pesticides.

The water samples were prepared and analyzed sequentially for endosulfan, heptachlor, dicofol, p,p′-DDT, aldrin, chlorpyrifos, and diazinon according to the extraction technique for aqueous matrix was carried out according to Edgell and Wesselman^20^ to analyze semi-volatile and non-volatile organic compounds. Water samples (500 mL) were transferred into a 1.000 mL separatory funnel. The samples were extracted thrice with 100 mL portions of 1:1 (v/v) ethyl acetate dichloromethane. The separatory funnel was clapping for 30 min to allow phase separation. The combined organic phases were collected into a 500 mL beaker, with the aqueous phase discarded. The combined organic layer was dried from the aqueous substance with 20 g of anhydrous sodium sulfate and allowed to settle. The organic content was decanted into a 300 mL round bottom flask, and the content was evaporated to dryness using the rotary evaporator at 40 °C. The pesticide was dissolved and collected with 2 mL of ethyl acetate and transferred into a 2 mL vial to be ready for cleanup^21^.

### Fish samples and analysis

Two fish species (Nile tilapia and African catfish) were caught from the Kitchener drain during the four seasons (Table 1). Twelve fish specimens of each were collected per season, washed with deionized water, put in cleaned plastic bags, and stored frozen until analysis was carried out. A 20 g sample of fish muscles was weighed into a 150 mL conical flask, and then 20 g and 5 g of sodium hydrogen carbonate and anhydrous sodium sulfate were added, respectively. The fish samples were added to 100 mL of the 1:1 (v/v) ethyl acetate: dichloromethane combination mixed well by shaking the conical flask while it was corked. The conical flask's contents were then supplemented with 20 g of anhydrous sodium sulfate and 20 g of sodium hydrogen carbonate. The mixture was agitated vigorously for 10 min while tightly corked the conical flask. Three hours were given for the content to stand. The organic layer was evaporated at 40 °C after being decanted into a 200 mL round bottom flask. The pesticide in the rotary flask was dissolved, collected with 2 mL of ethyl acetate, and transferred into a 2 mL vial to be ready for the cleanup^22^.

**Table 1:** The weight and length of fish species were assessed in this study (derived from Metwally et al.^7^).

		Nile tilapia	African catfish
Winter	Weight (g)	235.74 ± 44.85	626.40 ± 167.03
	Length (cm)	22.36 ± 1.98	39.50 ± 5.10
Spring	Weight (g)	94.18 ± 26.38	366.53 ± 88.23
	Length (cm)	19.35 ± 2.37	32.87 ± 4.43
Autumn	Weight (g)	130.04 ± 46.19	374.03 ± 107.45
	Length (cm)	16.91 ± 1.23	33.95 ± 6.54
Summer	Weight (g)	205.57 ± 56.43	567.27 ± 167.41
	Length (cm)	15.85 ± 1.92	28.91 ± 5.67

Values (± SD) with a total number of 12 fish per season.

### Assessment of human risk

The estimated daily (EDI) or weekly intake (EWI) of endosulfan, heptachlor, dicofol, p,p′-DDT, aldrin, chlorpyrifos, and diazinon by fish consumption were examined using the two equations below:

$${\text{EDI}} = \left( {{\text{Cm}} \times {\text{IR}}} \right)/{\text{BW}}$$

$${\text{EWI}} = {\text{EDI}} \times {7}$$

where, Cm represents the oregano chlorine concentrations in fish samples (μg/g-ww); IR is the daily intake rate of fish (62.25 g/person/day) and BW is the average body weight (15 kg for a child, 40 kg for a youth, and 70 kg for an adult)^23^.

$${\text{THQ}} = \left( {{\text{EF}} \times {\text{ED}} \times {\text{IR}} \times {\text{C}}} \right)/\left( {{\text{RfD}} \times {\text{WAB}} \times {\text{ATn }} \times {1}0^{{ - {3}}} } \right)$$

EF: the exposure frequency (days/year); ED : the exposure duration-years; IR: the ingestion rate -g/day; C: the metal concentration in fish (μg/kg); RfD: the oral reference dose (endosulfan = 0.006, heptochlor = 0.0005, dicofol = 0.0004, DDT = 0.0005, aldrin = 0.001, and chlorpyrifos = 0.0025) mg/kg/day; WAB: the average (kg) body weight, and ATn : the average exposure time (days/year × ED) for non-carcinogens.

The hazard index (HI) has been performed to assess the probable human health hazard of organochlorine and organophosphorus compounds. The HI refers to the sum of all THQ for various organochlorine and organophosphorus compounds exposures as shown in the equation below:

$$\begin{aligned} {\text{HI}} = & \Sigma {\text{TTHQs}} = {\text{THQ endosulfan}} + {\text{THQ heptochlor}} + {\text{THQ dicofol }} \\ & \quad + {\text{THQ DDT}} + {\text{THQ aldrin}} + {\text{THQ chlorpyrifos}} \\ \end{aligned}$$

where Σ TTHQs is the target hazard quotients of all organochlorine and organophosphorus compounds; while, the hazard index becomes over 1, the possible human health risk is expected^24^.

### Consent to participate

The authors are informed and agree to the study.

### Ethical approval

The ethical committee of the Faculty of Aquatic and Fisheries Sciences, Kafrelsheikh University approved the experimental protocol and all methods in the present study for treating animals for scientific purposes. All experiments were performed in accordance with relevant guidelines and regulations. Our reporting of research involving animals follows the recommendations of the ARRIVE guidelines.

## Results and discussion

### Organochlorine and organophosphorus compounds in water samples

In this study, the water samples were collected from Kitchener Drain (Table 2), and the results indicated that the heptachlor and diazinon were not detected during the four seasons. However, the levels of endosulfan were 29.7 ± 2.48 and 11.4 ± 1.48 ppb during the winter and autumn, respectively. Dicofol was also detected in the water during autumn and summer at 14.6 ± 3.19 and 33.2 ± 5.63 ppb, respectively. Only the DDT was detected during spring and showed a value of 10.8 ± 1.25 ppb. In summer, aldrin was only detected in the water samples at 21.1 ± 3.71 ppb. Chlorpyrifos was detected during winter and autumn at 12.1 ± 1.91 and 68.7 ± 7.83 ppb, respectively but did not detect during spring and summer in the Kitchener drain. The results are comparable with Shalaby et al.^15^, who stated that the levels of endosulfan (18.6–40 ppb), heptachlor (22.8–47.1 ppb), dicofol (17.4–31.8 ppb), p,p′-DDT (25.8–88.3 ppb), and chlorpyrifos (37–53.8 ppb) were detected in the water samples collected from the river Nile during the four seasons. According to the European Water Framework Directive (WFD, 2000/60/EC)^25^, the detected pesticide levels in this study are not significantly harmful and toxic. Several factors affect the drainage water in big drains, such as the effluents of agricultural and industrial wastes and untreated sewage water^26^. Kitchener Drain receives drainage from several agricultural drains containing pesticides and insecticides^13^. Organochlorine and organophosphorus based pesticides are heavily used in agriculture, but their derivates could pollute the water bodies and cause severe impacts^10^. The pesticides are applied in the agriculture sector, where the water runoff can affect the dilution of organochlorine and organophosphorus compounds^3^. Indeed, soil erosion and the level of suspended derivatives, including organochlorine and organophosphorus compounds, are strongly affected by rainfall events^27^. Hence, the high flood rate of water during the summer results in high dilution for the organochlorine and organophosphorus compounds^15^ and, thereby, low presence in the water and fish samples.

**Table 2:** Organochlorine and organophosphorus pesticides in the collected water samples (ppb) from Kitchener Drain during the four seasons.

Pesticides	Winter	Spring	Autumn	Summer
Endosulfan	29.7 ± 2.48	ND	11.4 ± 1.48	ND
Heptachlor	ND	ND	ND	ND
Dicofol	ND	ND	14.6 ± 3.19	33.2 ± 5.63
p,p′-DDT	ND	10.8 ± 1.25	ND	ND
Aldrin	ND	ND	ND	21.1 ± 3.71
Chlorpyrifos	12.1 ± 1.91	ND	68.7 ± 7.83	ND
Diazinon	ND	ND	ND	ND

Values (± SD) (n = 3). *ND* Not detected.

### Organochlorine and organophosphorus compounds in fish muscles

Table 3 shows the levels of analyzed organochlorine and organophosphorus compounds in Nile tilapia and catfish collected from the Kitchener drain during four seasons.

**Table 3:** Organochlorine and organophosphorus pesticides in the muscle samples (ppb) of Nile tilapia and African catfish from Kitchener Drain during the four seasons.

Fish	Season	Endosulfan	Heptachlor	Dicofol	p,p′-DDT	Aldrin	Chlorpyrifos	Diazinon	Total
Nile tilapia	Winter	15.2 ± 4.15	16.4 ± 5.15	ND	ND	14.03 ± 2.65	ND	ND	45.63
	Spring	ND	20.8 ± 4.87	ND	ND	12.2 ± 1.9	ND	ND	33
	Autumn	35.6 ± 6.95	6.3 ± 1.73	21.3 ± 6.53	15.3 ± 5.56	ND	10.8 ± 3.92	17.5 ± 4.53	106.8
	Summer	ND	ND	24.3 ± 5.92	16.6 ± 3.42	ND	ND	ND	40.9
Total		50.8	43.5	45.6	31.9	26.23	10.8	17.5	
African catfish	Winter	19.21 ± 3.95	10.93 ± 1.68	9.6 ± 1.23	10.10 ± 1.63	ND	ND	ND	49.84
	Spring	21.3 ± 3.95	10.6 ± 1.51	ND	ND	8.23 ± 3.1	ND	ND	40.13
	Autumn	10.70 ± 2.65	13.85 ± 2.64	5.5 ± 1.71	21.50 ± 6.54	16.51 ± 4.96	4.70 ± 0.7	6.6 ± 0.073	79.36
	Summer	13.60 ± 3.61	2.60 ± 0.7	10.65 ± 2.85	5.70 ± 0.83	11.6 ± 4.18	3.30 ± 0.02	11.80 ± 1.31	59.25
Total		64.81	37.98	25.75	37.3	36.34	8	18.4	
Permitted levels	FAO^28^	100–200	200	–	500	200	1000	200	–
	USFDA^29^	–	300	–	300	300	–	–	–
	CFIA^30^	–	5	–	5000	100	–	500	–
	FSANZ^31^	–	5	–	1000	100	–	500	–

Values (± SD) (n = 3). *ND* Not detected.

In Nile tilapia, endosulfan (15.2 ± 4.15 ppb), heptachlor (16.4 ± 5.15 ppb), and aldrin (14.03 ± 2.65 ppb) were detected during the winter season. Only heptachlor (20.8 ± 4.87 ppb) and aldrin (12.2 ± 1.9 ppb) were detected in Nile tilapia during spring. In the autumn season, endosulfan (35.6 ± 6.95 ppb), heptachlor (6.3 ± 1.73 ppb), dicofol (21.3 ± 6.53 ppb), p,p′-DDT (15.3 ± 5.56 ppb), chlorpyrifos (10.8 ± 3.92 ppb), and diazinon (17.5 ± 4.53 ppb) were detected in Nile tilapia. In summer, dicofol (24.3 ± 5.92 ppb) and p,p′-DDT (16.6 ± 3.42 ppb) were detected, while endosulfan, heptachlor p,p′-DDT, aldrin, chlorpyrifos, and diazinon were not detected in Nile tilapia. In line with the current study, Shalaby et al.^15^ stated that pesticides were also detected in tilapia collected from the river Nile, Cairo, Egypt, during the four seasons. Generally, the levels of endosulfan showed the highest detected pesticides with a total of 50.8 ppb in Nile tilapia during the four seasons, which is within the permitted levels as indicated by FAO^28^. Endosulfan is commonly used in agriculture, but the high accumulated levels cause endocrine disruption involved in fish growth and development. The detected heptachlor is 43.5 ppb which is within the permitted levels as reported by FAO^28^ and USFDA^29^, while it is over the permitted levels reported by CFIA^30^ and FSANZ^31^. The total detected p,p′-DDT (31.9 ppb), and aldrin (26.23 ppb) during the four seasons are within the accepted levels as indicated by FAO^28^, USFDA^29^, CFIA^30^, and FSANZ^31^. Chlorpyrifos showed a total of 10.8 ppb during the four seasons, which is considered a safe level as indicated by FAO^28^, while diazinon showed a total of 17.5 ppb, which is also safe compared to the reports of FAO^28^, CFIA^30^, and FSANZ^31^. It is worth noting that the highest organochlorine and organophosphorus compounds have been detected in Nile tilapia in autumn (106.8 ppb), followed by winter (45.63 ppb), then summer (40.9 ppb) and spring (33 ppb), respectively. This might be as a result of the high hydrophobicity and lipophilicity of these compounds as well as their potential retention in organisms' organic phases^32^.

In African catfish, endosulfan (19.21 ± 3.95 ppb), heptachlor (10.93 ± 1.68 ppb), dicofol (9.6 ± 1.23 ppb), and p,p′-DDT (10.10 ± 1.63) were detected during the winter season while chlorpyrifos, aldrin, and diazinon were not detected. In the spring season, endosulfan (21.3 ± 3.95 ppb), heptachlor (10.6 ± 1.51 ppb), and aldrin (8.23 ± 3.1 ppb) were detected in African catfish. In the autumn season, endosulfan (10.70 ± 2.65 ppb), heptachlor (13.85 ± 2.64 ppb), dicofol (5.5 ± 1.71 ppb), p,p′-DDT (21.50 ± 6.54 ppb), aldrin (16.51 ± 4.96 ppb), chlorpyrifos (4.70 ± 0.7 ppb), and diazinon (6.6 ± 0.073 ppb) were detected in African catfish. Similarly, in the summer season, endosulfan (13.60 ± 3.61 ppb), heptachlor (2.60 ± 0.7 ppb), dicofol (10.65 ± 2.85 ppb), p,p′-DDT (5.70 ± 0.83 ppb), aldrin (11.6 ± 4.18 ppb), chlorpyrifos (3.30 ± 0.02 ppb), and diazinon (11.80 ± 1.31 ppb) were detected in African catfish. The analyzed pesticides are in line with Shalaby et al.^15^ who stated that pesticides derivatives were detected in African catfish collected from the river Nile, Cairo, Egypt, during the four seasons. In the same manner as Nile tilapia, endosulfan showed the highest detected level (64.81 ppb) in African catfish during the four seasons but still within the permitted levels, as reported by FAO^28^. The detected heptachlor is 37.98 ppb which is within the permitted levels as reported by FAO^28^ and USFDA^29^, while it is over the permitted levels reported by CFIA^30^ and FSANZ^31^. The total detected p,p′-DDT (37.3 ppb), and aldrin (36.34 ppb) during the four seasons are within the accepted levels as indicated by FAO^28^, USFDA^29^, CFIA^30^, and FSANZ^31^. Chlorpyrifos showed a total of 8 ppb during the four seasons, which is considered a safe level as indicated by FAO^28^, while diazinon showed a total of 18.4 ppb, which is also safe compared to the reports of FAO^28^, CFIA^30^, and FSANZ^31^. The detected pesticides in African catfish followed the sequence of autumn (79.36 ppb) > summer (59.25 ppb) > winter (49.84 ppb) > spring (40.13 ppb). Shalaby et al.^15^ reported similar results where the pesticides residues showed the same manner (autumn > summer > winter > spring) in African catfish and tilapia collected from the river Nile, Cairo, Egypt. Both fish species are potential targets for organochlorine and organophosphorus compounds accumulation from the pesticides polluted water. In African catfish, the high-fat content allows the soluble lipids organochlorine and organophosphorus compounds to accumulate massively in edible fish tissue causing severe pollution.

### Estimated organochlorine and organophosphorus pesticides

The accumulation of pesticides in fish affects the food chain through daily intake^33^. Hence, this study evaluated the estimated daily intakes (EDI) of pesticides in Nile tilapia and African catfish collected from the Kitchener drain. Several organizations report the permissible daily intake (PDI) of pesticides in fish to compare with EDI and predict the possibility of human risk^34, 35^. The data in Table 4 indicates that the EDI of endosulfan (0–47.66 μg/kg BW/day), heptachlor (0–0.0018 μg/kg BW/day), dicofol (0–0.021 μg/kg BW/day), p,p′-DDT (0–0.013 μg/kg BW/day), aldrin (0–0.012 μg/kg BW/day), chlorpyrifos (0–0.009 μg/kg BW/day), and diazinon (0–0.015 μg/kg BW/day) in Nile tilapia collected during the four seasons. However, the EDI during the winter (for dicofol, p,p′-DDT, chlorpyrifos, and diazinon), during the spring (for endosulfan, dicofol, p,p′-DDT, aldrin, chlorpyrifos, and diazinon), during autumn (for aldrin), and during summer (for heptachlor, aldrin, chlorpyrifos, and diazinon) recorded 0 μg/kg BW/day. The sequence of EDI in Nile tilapia during the four seasons is heptachlor > endosulfan > dicofol > p,p′-DDT > aldrin > diazinon > chlorpyrifos. The results also indicate that the EDI in Nile tilapia collected from the Kitchener drain during the four seasons is lower than the PDI according to codex Alimentarius^34^ and FDA^35^ (μg/kg bwt). Further, the results are similar to Barakat et al.^36^ and Shalaby et al.^15^, who stated that the EDI of Nile tilapia collected from Lake Qarun and Nile River in Cairo were not over the PDI.

**Table 4:** The estimated daily intakes (EDI) of pesticides (μg/kg BW/day) of Nile tilapia and African catfish from Kitchener Drain during the four seasons by adult people (70 kg per person).

Fish	Season	Endosulfan	Heptachlor	Dicofol	p,p′-DDT	Aldrin	Chlorpyrifos	Diazinon
Nile tilapia	Winter	0.013	0.014	0	0	0.012	0	0
	Spring	0	0.018	0	0	0.010	0	0
	Autumn	0.047	0.05	0.018	0.013	0	0.009	0.015
	Summer	0.009	0	0.021	0.012	0	0	0
Total		0.069	0.082	0.039	0.025	0.022	0.009	0.015
African catfish	Winter	0.017	0.009	0.008	0.009	0	0	0
	Spring	0.018	0.009	0	0	0.007	0	0
	Autumn	0.009	0.012	0.004	0.019	0.014	0.004	0.005
	Summer	0.012	0.002	0.009	0.005	0.010	0.002	0.010
Total		0.056	0.032	0.021	0.033	0.031	0.006	0.015
PDI^1^		6	0.1		100	0.1		
PDI^2^			300			300		

^1^Permissible daily intake (PDI, μg/kg bwt) according to codex Alimentarius^34^.

^2^The PDI according to FDA tolerance or critical limit for human consumption of fish^35^.

In African catfish, the EDI of endosulfan (0.009–0.018 μg/kg BW/day), heptachlor (0.002–0.012 μg/kg BW/day), dicofol (0–0.009 μg/kg BW/day), p,p′-DDT (0–0.019 μg/kg BW/day), aldrin (0–0.014 μg/kg BW/day), chlorpyrifos (0–0.002 μg/kg BW/day), and diazinon (0–0.01 μg/kg BW/day) during the four seasons. Nevertheless, the EDI during the winter (for aldrin, chlorpyrifos, and diazinon) and during the spring (for dicofol, p,p′-DDT, chlorpyrifos, and diazinon) recorded 0 μg/kg BW/day. The sequence of EDI in African catfish during the four seasons is endosulfan > p,p′-DDT > heptachlor > aldrin > dicofol > diazinon > chlorpyrifos. The results also indicate that the EDI in African catfish collected from the Kitchener drain during the four seasons is lower than the PDI^34^ and FDA^35^. Similarly, Shalaby et al.^15^ stated that the EDI of African catfish collected from the Nile River in Cairo was not over the PDI.

In Nile tilapia or African catfish, the differences in the EDI during the four seasons can be altered by the surface runoff of water and levels of suspended pesticides. Concisely, the precipitation rate of pesticides may differ as a response to seasonality effects on the surface water runoff during rainy and dry seasons^37^. In addition, the consumed pesticides in agriculture is a season-dependent practice that may affect the presence of specific pesticide residuals in the surface waters^38^.

### Target hazard quotient

The values of the target hazard quotients (THQ) refer to the possible health risk for the pesticides, where a value over one indicates health risk^39^. In this study, the THQ for Nile tilapia and African catfish collected from Kitchener drain less than one in the case of all detected organochlorine and organophosphorus compounds during the four seasons (Table 5). Besides, the hazard index (HI) recorded less than one for all organochlorine and organophosphorus compounds in the Nile tilapia and African catfish collected during the four seasons. The results are in line with Shalaby et al.^15^, Barakat et al.^36^, and Abbassy et al.^32^, who stated that no hazardous effects for pesticides in the fish collected from Nile River in Cairo, Lake Qarun, and Edko lake, respectively. Usually, health problems could emerge from the persistent pesticide accumulation in human body tissues due to consuming polluted fish^32^.

**Table 5:** Target hazard quotient (THQ) for pesticides in Nile tilapia and African catfish from Kitchener Drain during the four seasons.

Fish	Season	Target Hazard Quotient (THQ)							Hazard index (HI)	Human risk
		Endosulfan	Heptachlor	Dicofol	p,p′-DDT	Aldrin	Chlorpyrifos	Diazinon		
Nile tilapia	Winter	0.0022	0.029	0	0	0.024	0	0	0.056	No
	Spring	0	0.036	0	0	0.021	0	0	0.058	No
	Autumn	0.0052	0.011	0.047	0.027	0	0.009	0.006	0.106	No
	Summer	0.0016	0	0.054	0.024	0	0	0	0.079	No
African catfish	Winter	0.002	0.018	0.021	0.017	0	0	0	0.060	No
	Spring	0.003	0.018	0	0	0.014	0	0	0.036	No
	Autumn	0.0015	0.024	0.012	0.038	0.029	0.004	0.002	0.112	No
	Summer	0.002	0.004	0.023	0.010	0.020	0.002	0.004	0.068	No

## Conclusion

The results concluded that endosulfan, heptachlor, dicofol, p,p′-DDT, aldrin, chlorpyrifos, and diazinon were detected in the water of Kitchener drain but in a season-dependent manner. It is worth noting that the highest organochlorine and organophosphorus compounds have been detected in Nile tilapia in autumn, followed by winter, then summer and spring. In addition, organochlorine and organophosphorus compounds were detected in African catfish following the sequence of autumn > summer > winter > spring. The estimated daily intake for all detected organochlorine and organophosphorus compounds was below the referenced permissible daily intake in Nile tilapia and African catfish. Besides, the results confirmed the absence of a hazard index for consuming Nile tilapia and African catfish collected from the Kitchener drain. However, future studies are suggested to confirm the results obtained by considering having more sampling sites along the Kitchener drain.

## Data Availability

Data are available upon request from the corresponding author.

## References

[1] Li Z (2022). Prioritizing agricultural pesticides to protect human health: A multi-level strategy combining life cycle impact and risk assessments. Ecotoxicol. Environ. Saf..

[2] Parra-Arroyo L, González-González RB, Castillo-Zacarías C (2022). Highly hazardous pesticides and related pollutants: Toxicological, regulatory, and analytical aspects. Sci. Total Environ..

[3] Tudi M, Li H, Li H (2022). Exposure routes and health risks associated with pesticide application. Toxics..

[4] Sabzevari S, Hofman J (2022). A worldwide review of currently used pesticides' monitoring in agricultural soils. Sci. Total Environ..

[5] Fu H, Tan P, Wang R (2022). Advances in organophosphorus pesticides pollution: Current status and challenges in ecotoxicological, sustainable agriculture, and degradation strategies. J. Hazard. Mater..

[6] Prajapati S, Challis JK, Jardine TD, Brinkmann M (2022). Levels of pesticides and trace metals in water, sediment, and fish of a large, agriculturally-dominated river. Chemosphere.

[7] Metwally AA, Khalafallah MM, Dawood MAO (2023). Assessment of the water quality, the human health risk, and heavy metal accumulation in Nile tilapia and African catfish collected from the Kitchener Drain-Egypt. Reg. Stud. Mar. Sci..

[8] Slaby S, Le Cor F, Dufour V (2022). Distribution of pesticides and some of their transformation products in a small lentic waterbody: Fish, water, and sediment contamination in an agricultural watershed. Environ. Pollut..

[9] Olisah C, Okoh OO, Okoh AI (2020). Occurrence of organochlorine pesticide residues in biological and environmental matrices in Africa: A two-decade review. Heliyon..

[10] Sultan M, Hamid N, Junaid M, Duan J-J, Pei D-S (2023). Organochlorine pesticides (OCPs) in freshwater resources of Pakistan: A review on occurrence, spatial distribution and associated human health and ecological risk assessment. Ecotoxicol. Environ. Saf..

[11] Dahshan H, Megahed AM, Abd-Elall AMM, Abd-El-Kader MA-G, Nabawy E, Elbana MH (2016). Monitoring of pesticides water pollution-the Egyptian river Nile. J. Environ. Health Sci. Eng..

[12] El-Alfy MA, Hasballah AF, Abd El-Hamid HT, El-Zeiny AM (2019). Toxicity assessment of heavy metals and organochlorine pesticides in freshwater and marine environments, Rosetta area, Egypt using multiple approaches. Sustain. Environ. Res..

[13] Abd-Elfattah EA, Sheta AEA, Saifeldeen M, Hassanein SA, Mahmoud YI (2021). Assessment of water and sediments quality of Kitchener Drain Nile Delta-Egypt. Arab Univ. J. Agric. Sci..

[14] FAO. World Fisheries and Aquaculture. Food and Agriculture Organization, Rome, 10.4060/cc0461en.2022 (2022).

[15] Shalaby SEM, El-Saadany SS, Abo-Eyta AM, Abdel-Satar AM, Al-Afify ADG, Abd El-Gleel WMM (2018). Levels of pesticide residues in water, sediment, and fish samples collected from Nile River in Cairo, Egypt. Environ. Forensics.

[16] Intisar A, Ramzan A, Sawaira T (2022). Occurrence, toxic effects, and mitigation of pesticides as emerging environmental pollutants using robust nanomaterials–A review. Chemosphere.

[17] Mali H, Shah C, Raghunandan BH (2023). Organophosphate pesticides an emerging environmental contaminant: Pollution, toxicity, bioremediation progress, and remaining challenges. J. Environ. Sci..

[18] Mansour SA (2004). Pesticide exposure—Egyptian scene. Toxicology.

[19] Malhat F, Nasr I (2011). Organophosphorus pesticides residues in fish samples from the river Nile Tributaries in Egypt. Bull. Environ. Contam. Toxicol..

[20] Edgell, K.W., & Wesselman, R.J. USEPA (Environmental Protection Agency) method study 36 SW-846 methods 8270/3510, GC/MS (gas chromatography/mass spectrometry) method for semivolatile organics: Capillary-column technique separatory-funnel liquid-liquid extraction. Final report, September 1986–December 1987*.* United States 1989-04-01 (1989).

[21] Essumang D, Togoh G, Chokky L (2009). Pesticide residues in the water and fish (lagoon tilapia) samples from lagoons in Ghana. Bull. Chem. Soc. Ethiopia.

[22] Akan J, Mohammed Z, Jafiya L, Audu S (2013). Organophosphorus pesticide residues in different tissues of fish samples from Alau Dam, Borno State, Nigeria. World J. Fish Mar. Sci..

[23] EPA. Environmental Protection Agency, Integrated risk information system. Office of Health Environmental Assessment, Washington, DC (1996).

[24] Huang M, Zhou S, Sun B, Zhao Q (2008). Heavy metals in wheat grain: Assessment of potential health risk for inhabitants in Kunshan, China. Sci. Total Environ..

[25] WFD. Water Framework Directive 2000/60/EC, “Directive 2000/60/EC of the European Parliament and of the Council of 23 October 2000 establishing a framework for Community action in the field of water policy”, https://eur-ex.europa.eu/legalcontent/EN/TXT/PDF/?uri=CELEX:32000L0060&from=EN (2000).

[26] Arisekar U, Shakila RJ, Shalini R (2022). Bioaccumulation of organochlorine pesticide residues (OCPs) at different growth stages of pacific white leg shrimp (*Penaeus vannamei*): First report on ecotoxicological and human health risk assessment. Chemosphere.

[27] Ashesh A, Singh S, Linthoingambi Devi N, Chandra YI (2022). Organochlorine pesticides in multi-environmental matrices of India: A comprehensive review on characteristics, occurrence, and analytical methods. Microchem. J..

[28] FAO. Fao/who's codex alimentarius commission. Report the meeting of fao and non-fao regional fishery bodies on arrangements. Rome, Italy (1999).

[29] USFDA (2001). Food code, 2001 recommendation of the United States public health service, food and drug administration. Natl. Inf. Serv. Publ. PB.

[30] CFIA. Canadian food inspection agency. Risk assessment phpa request: 2005–07 decision document (2005).

[31] FSANZ. Food standards Australia and New Zealand 206. Available: http://web2.warillanh.schools.nsw.edu.au/text_books/pdhpe/PDHE_in_Focus/yr11/online_book/resources/pdf/chapter_01/food_comp.pdf (2006).

[32] Abbassy MA, Khalifa MA, Nassar AMK, El-Deen EEN, Salim YM (2021). Analysis of organochlorine pesticides residues in fish from Edko Lake (North of Egypt) using eco-friendly method and their health implications for humans. Toxicol. Res..

[33] Yohannes YB, Ikenaka Y, Saengtienchai A, Watanabe KP, Nakayama SMM, Ishizuka M (2014). Concentrations and human health risk assessment of organochlorine pesticides in edible fish species from a Rift Valley lake–Lake Ziway, Ethiopia. Ecotoxicol. Environ. Saf..

[34] CAC. Codex Alimentarius Commission. FAO/WHO Food STDs Codex Alimentarius pesticide residues in food–MRLs & Extraneous MRLs last updated March. http://www.codexalimentarius.net/mrls/pestdes/jsp/pest_q-e.jsp. Accessed September 2020. (2009).

[35] FDA. Food and Drug Administration. Fish and Fisheries Products Hazards and Controls Guidance. 4th ed. Chapter 9. Center for Food Safety and Applied Nutrition, US Food and Drug Administration (2011).

[36] Barakat AO, Khairy M, Aukaily I (2017). Bioaccumulation of organochlorine contaminants in fish species from Lake Qarun, a protected area of Egypt. Toxicol. Environ. Chem..

[37] Kruć-Fijałkowska R, Dragon K, Drożdżyński D, Górski J (2022). Seasonal variation of pesticides in surface water and drinking water wells in the annual cycle in western Poland, and potential health risk assessment. Sci. Rep..

[38] Chaka B, Osano AM, Wesley ON, Forbes PBC (2023). Seasonal variation in pesticide residue occurrences in surface waters found in Narok and Bomet Counties, Kenya. Environ. Monit. Assess..

[39] Kalipci E, Cüce H, Ustaoğlu F, Dereli MA, Türkmen M (2023). Toxicological health risk analysis of hazardous trace elements accumulation in the edible fish species of the Black Sea in Türkiye using multivariate statistical and spatial assessment. Environ. Toxicol. Pharmacol..

